# Seaweed Protein Hydrolysates and Bioactive Peptides: Extraction, Purification, and Applications

**DOI:** 10.3390/md19090500

**Published:** 2021-08-31

**Authors:** Javier Echave, Maria Fraga-Corral, Pascual Garcia-Perez, Jelena Popović-Djordjević, Edina H. Avdović, Milanka Radulović, Jianbo Xiao, Miguel A. Prieto, Jesus Simal-Gandara

**Affiliations:** 1 Nutrition and Bromatology Group, Analytical and Food Chemistry Department, Faculty of Food Science and Technology, Ourense Campus, University of Vigo, E-32004 Ourense, Spain; javier.echave@uvigo.es (J.E.); mfraga@uvigo.es (M.F.-C.); pasgarcia@uvigo.es (P.G.-P.); jianboxiao@uvigo.es (J.X.); 2 Centro de Investigação de Montanha (CIMO), Instituto Politécnico de Bragança, Campus de Santa Apolonia, 5300-253 Bragança, Portugal; 3 Department of Chemistry and Biochemistry, Faculty of Agriculture, University of Belgrade, 11080 Belgrade, Serbia; jelenadj@agrif.bg.ac.rs; 4 Department of Science, Institute for Information Technologies Kragujevac, University of Kragujevac, 34000 Kragujevac, Serbia; edina.avdovic@pmf.ac.rs; 5 Department of Bio-Medical Sciences, State University of Novi Pazar, Vuka Karadžića bb, 36300 Novi Pazar, Serbia; mradulovic@np.ac.rs; 6 International Research Center for Food Nutrition and Safety, Jiangsu University, Zhenjiang 212013, China

**Keywords:** seaweed, protein, extraction, bioactive peptides, industrial application

## Abstract

Seaweeds are industrially exploited for obtaining pigments, polysaccharides, or phenolic compounds with application in diverse fields. Nevertheless, their rich composition in fiber, minerals, and proteins, has pointed them as a useful source of these components. Seaweed proteins are nutritionally valuable and include several specific enzymes, glycoproteins, cell wall-attached proteins, phycobiliproteins, lectins, or peptides. Extraction of seaweed proteins requires the application of disruptive methods due to the heterogeneous cell wall composition of each macroalgae group. Hence, non-protein molecules like phenolics or polysaccharides may also be co-extracted, affecting the extraction yield. Therefore, depending on the macroalgae and target protein characteristics, the sample pretreatment, extraction and purification techniques must be carefully chosen. Traditional methods like solid–liquid or enzyme-assisted extraction (SLE or EAE) have proven successful. However, alternative techniques as ultrasound- or microwave-assisted extraction (UAE or MAE) can be more efficient. To obtain protein hydrolysates, these proteins are subjected to hydrolyzation reactions, whether with proteases or physical or chemical treatments that disrupt the proteins native folding. These hydrolysates and derived peptides are accounted for bioactive properties, like antioxidant, anti-inflammatory, antimicrobial, or antihypertensive activities, which can be applied to different sectors. In this work, current methods and challenges for protein extraction and purification from seaweeds are addressed, focusing on their potential industrial applications in the food, cosmetic, and pharmaceutical industries.

## 1. Introduction

Seaweeds are considered an important source of macronutrients, especially proteins and lipids, and micronutrients, represented by vitamins and minerals, together with dietary fiber and other minoritarian constituents, as it is the case of polyphenols. This rich variety of biomolecules turns macroalgae into a well appreciated resource for the extraction of natural ingredients aimed to the development of nutraceuticals, functional food, cosmetics, pharmaceutical products, or animal feeding, among others [1]. Furthermore, the estimated rise of the global population for 2050 is an international concern since it is expected a parallel increase of protein demand. In this sense, seaweed may stand for a potential source of proteins [2]. Indeed, macroalgae have been reported to annually produce higher protein yield per surface area (2.5–7.5 annual tons per hectare) than terrestrial plants (around 1 to 2 tons per hectare for soybean, legumes, or wheat) [3]. The protein content in seaweeds is variable among red (Rhodophyta), brown (Ochrophyta), and green (Chlorophyta) seaweeds. For instance, red seaweeds are considered the most prominent source of proteins, with protein content representing between 19 and 44% of dry weight (dw), while the green and brown ones exhibit lower protein amounts, around 20% or 10% of dry weight, respectively [4]. These values are comparable and even slightly higher than those of legumes (20–30%), cereals (10–15%), or nuts (20–30%) [5].

As previously reported, the protein concentration in algal sources depends on several factors, including interspecific variations, geographical location, environmental conditions, and seasonal variations [6]. The maximal protein contents have been described between winter and the beginning of spring, while minimal levels were reported by summer and early fall [7]. Besides, macroalgae, as it occurs with terrestrial plants, are highly susceptible to the presence of different biotic and abiotic stress signals, which contribute to the regulation of protein expression and the biosynthesis of specialized metabolites.

Regarding the variability of the protein profile of macroalgae, they show a rich source of several types of seaweed protein (SP) and derivatives—such as peptides, enzymes, glycoproteins, lectins, and mycosporine-like amino acids (MAAs), as well as phycobiliproteins, characteristic of red seaweeds [4]. Understanding the quality of proteins, in terms of amino acid composition and digestibility, is a fundamental step facing their use for human consumption. Thus, concerning amino acid composition, proteins of high quality are those holding high proportions of essential amino acids (EAAs), due to the impossibility of being synthesized by the human body. In the case of SP, the most abundant amino acids are glycine, alanine, arginine, proline, glutamic, and aspartic acids, while tryptophan, cysteine and lysine are present in a lower extent. The sum of aspartic and glutamic acids content in macroalgae may stand for about 30% of the total amino acids (Table 1). Consequently, the combination of macroalgae ingest with other protein-enriched foods is regarded as an optimal approach for a high-quality intake of proteins [8]. On the other hand, protein quality depends on their digestibility. Indeed, the higher proportion of poorly digestible amino acids the lower nutritional value of SP. Besides, digestibility can be altered by distinct factors, such as the presence of anti-nutritional molecules like some polysaccharides or tannin derivates, among others [3,8]. In recent years, scientific works have analyzed both the amino acids profile and the digestibility of macroalgal proteins to accurately predict their nutritional quality (Table 1). In general terms, algal proteins display a rich free amino acid profile and remarkable digestibility rates, mostly higher than 70% and thus, comparable to those of grains (69–84%), legumes (72–92%), fruits (72–92%), and vegetables (68–80%) [3].

Therefore, macroalgae stand for a sustainable source of proteins that can be applied as ingredients for human and animal consumption. In fact, seaweed have been consumed since ancient times, mostly in Asian countries, and nowadays, they may be used for fortifying food or feed matrixes either with low protein content or poor amino acidic profile. They can be also used as natural food preservatives or additives to improve the organoleptic properties of food products while minimizing the side effects associated to their synthetic analogues [9]. Indeed, food grade phycobiliproteins are generally recognized as safe (GRAS), which point them as target compounds for their direct application in food industry.

For instance, phycobiliproteins from *Neoporphyra haitanensis* (formerly *Porphyra haitanensis*) were investigated for their further inclusion in liposome–meat systems. They have a high EAA content (43%) and significant antioxidant capacity, able to reduce lipid peroxidation [16]. In the same way, SP have been involved in the development of functional products, as reported for the protein hydrolysates from *Palmaria palmata*, which were incorporated into bread to keep its renin inhibitory activity after baking, thus preserving its cardiovascular protective properties. Hence, the fortification of commonly consumed products makes SP excellent carriers of bioactive compounds, showing a wide range of health benefits [17].

In this sense, several biological activities have been associated to SP, hydrolysates, or peptides that are of great interest for other industrial sectors, such as pharmacology or cosmetics. SP can be found inside cell cytoplasm and/or attached to macroalgae cell wall polysaccharides (e.g., ulvan, alginate, carrageenan…) forming diverse complexes. These polymers are mostly composed of glucans of different nature depending on the species, and preeminently xylan-based polysaccharides, which are highly resistant to hydrolysis [18]. Hence, disrupting these cell wall polysaccharides is an essential process that must be conducted to obtain and further process SP. To obtain SP hydrolysates, these proteins must be degraded either by physical (energy) or chemical methods (e.g., endoproteases, pH-induced degradation). For example, *P. palmata,* was used to obtain a protein hydrolysate with the ability of improving glycemia and insulin production when assessed in vivo, as the specific Alcalase/Flavourzyme protein hydrolysate enhanced the glucose tolerance and satiety. Thus, these *P. palmata* protein hydrolysates were suggested as potential molecules for managing two chronic diseases with a worldwide increasing prevalence: obesity and type-2 diabetes mellitus [19]. Another work based on proteins extracted from the same species proved their antiproliferative capacity against five human cancer cell lines derived from breast (MCF-7), liver (HepG-2), gastric (SGC-7901), lung (A549), and colon cancers (HT-29), with half inhibitory concentrations (IC_50_) ranging between 192 and 317 μg/mL [20]. In the field of cosmetics, MAAs are of great interest for the formulation of sunscreens, thanks to their low molecular weight, hydrosolubility, and chemical stability towards light and heat. Arginine, which can be found at higher concentrations in *Palmaria* and *Porphyra* species, is also a highly appreciated amino acid in cosmetics for being a precursor of urea, a widely used cosmetic ingredient [21].

Therefore, macroalgae represent a sustainable and natural source of high-quality proteins and protein-derived molecules. Their multiple applications as food, nutraceutical or drug ingredients revealed them as cost-effective and profitable molecules for their use in various industries. Moreover, SP hydrolysates have displayed increased bioactive properties in comparison to whole proteins. Herein, available methods of SP extraction, purification, and hydrolysate production will be discussed in the following sections.

## 2. Extraction Technologies

Prior to hydrolysate or peptide production, SP must be released and isolated from the rest of biomass components, which requires disruption of the seaweed cell wall polysaccharides. This implies that macroalgae biomass must be subjected to a pretreatment stage prior extraction, involving different disruptive techniques, that would aid to improve the extraction yield. A combinatorial approach including a coordinate pretreatment and extraction technique increases protein recovery [22]. Pretreatment methods aim at breaking cell walls to release the intracellular fraction of biological samples, including free and cell wall-attached proteins, depending on the system of choice. Extensively used pretreatment methods thus include mechanical grinding, osmotic shock (OS), alkaline treatment, freeze-thaw, or ultrasonic sonication [4]. Since seaweeds are marine organisms, they are susceptible to strong osmotic pressures, and their cell walls may be broken by allowing the seaweed biomass to be transferred into hypotonic solutions. This may be achieved using ultrapure or de-ionized water to induce OS [23]. In the same manner, a combination of sonication and OS has been proven to increase SP yield and extractability [24]. Nonetheless, one of the most valuable pretreatment methods involves the application of glucanases to the extraction mix to maximize SP yield [25]. Using fresh, dried or freeze-dried seaweed samples may also influence the resulting SP yield, although yield variations are not significant. Drying tests on several *Sargassum* species revealed that freeze-dried seaweeds subjected to classical SLE yielded slightly more extractable protein in comparison to oven-dried [26]. However, Angell et al. found out that using fresh pulped *Ulva ohnoi* blades yielded as much as by two-fold the extractable proteins, applying the same SLE method, but following a sample management closer to that of terrestrial plants [27]. In the same manner, heat treatment exerts a differential effect on SP extractability depending on the algal biomass. For example, in the study carried out by O’ Connor et al., autoclave treatment (>121 °C) resulted in higher SP yields for *P. palmata* but not for *F. vesiculosus* or *Alaria esculenta* [24]. In summary, diverse pretreatment and extraction options must be specifically studied and assessed for each seaweed species, reaching meaningful rates of SP yield, stability, or digestibility.

Extraction methods currently available for protein extraction include solid–liquid extraction (SLE), enzyme-assisted extraction (EAE), pulse-electric field (PEF), high hydrostatic pressure extraction (HHPE), ultrasound-assisted extraction (UAE), and microwave-assisted extraction (MAE). In general terms, solubilization is a paramount factor that modulates protein extraction, which depends on different physicochemical conditions. In fact, proteins can be extracted by their solubilization at different pH values, involving the sequential use of differently buffered solutions. Nevertheless, proteins are generally co-extracted with other interferents, such as sugar or phenolic compounds [25,28], forcing the application of protein precipitation to achieve the isolation and purification of the extracted SP. Moreover, except for EAE, extraction methods promote an unspecific protein hydrolysis, which is also accompanied by the liberation of intracellular proteases from cell walls that may further degrade protein structures. Protein integrity is also affected by the precipitation method of choice, aimed at reaching the protein isoelectric point (IP) by acidifying the medium pH and causing the ‘salting-out’ effect, improving protein solubility using salts like ammonium sulphate, (NH_4_)_2_SO_4_ [29]. Yet, protein stability is not a top priority feature for hydrolysate production. Thereafter, more disruptive methods may be used to that aim, such as MAE, PFE, or UAE.

### 2.1. Solid–Liquid Extraction

SLE is the most traditional method used for SP extraction, involving the use of different solvents, such as distilled water, buffered solutions, and lysis surfactant-containing solutions [30]. Nonetheless, about food applications, the use of either non-toxic or easy-to-remove reagents must be employed for SP extraction. Thus, demineralized and de-ionized water are the most extensively used solvents for the application of SLE methods, as they lead to performance of OS, helping protein extraction in a cost-effective and easy manner [31]. However, this method should be optimized to improve its efficiency, modulating different factors, such as the algal biomass/solvent ratio (w:v), temperature, or time.

Temperature is a key parameter in SP extraction, as it influences protein integrity, enzymatic activity, and solubility of other cellular constituents, such as cell wall polysaccharides [24]. Indeed, hot water has been classically selected to extract algal polysaccharides by SLE, also promoting the co-extraction of proteins. Therefore, SP aqueous extraction requires low temperatures (around 4 °C) to ensure protein integrity, whereas higher temperatures can be applied for protein hydrolysate production, thus involving heat-assisted extraction (HAE) or a combination with enzymatic pretreatments [32]. Due to the heterogeneous protein composition of algal extracts, SLE procedures are generally based on two sequential extraction procedures to meet the solubility requirements of different acidic and alkaline proteins, which includes an initial OS stage followed by the application of an alkaline NaOH solution [16,27]. The process is considered food-grade, as NaOH is used for protein extraction from various food matrices [28]. A reducing agent, normally 2-mercaptoethanol, is usually added to the alkaline solution to minimize potential protein degradation, although it has been reported as a toxic compound and has been increasingly replaced by N-acetyl-L-cysteine (NAC) for food purposes [22]. That shift on pH extraction has shown beneficial results on protein extraction, obtaining enhanced extraction yields, as it was seen on the combination of acidic SLE followed by alkaline SP extraction from *Ascophyllum nodosum* [33]. Moreover, the application of pH-shift combined with IP precipitation, reaching pH values between 2 and 4 ensures a maximum yield with this extraction method [29]. Combining these methods, a 14% protein yield (*w*/*w*) was obtained from *Porphyra dioica* [34]. Besides the combination of OS and an SLE extraction of alkaline-soluble proteins with NaOH, the use of a lysis solution holding urea, detergent and other reactants, allowed for extracting 11.8% (*w*/*w*) of protein from *Ulva* sp. [31]. While SP extraction yields by SLE may be near the total protein content of seaweed species, it is also a very time-consuming method [32].

### 2.2. Enzyme-Assisted Extraction

EAE is one of the most studied techniques used to disrupt macroalgal cell walls. The application of targeted polysaccharide-digesting enzymes, such as cellulases, hemicellulases, β-glucanases, and xylanases has been described as a food-grade approach to breakdown the macroalgae cell wall. Thus, commercial enzyme cocktails have been proven to be successful for this purpose [25]. However, seaweed cell wall composition can vary among phyla and species and, therefore, a right choice of carbohydrase(s), together with the optimization of operating conditions (enzyme:substrate ratio (E:S), temperature, pH) of individual enzymes or cocktails must be proved prior to large-scale extraction to maximize protein recovery. In general, EAE has been mostly studied on red and green seaweeds, because of their simpler composition with respect to brown seaweeds. A greater protein yield has been reported on *Solieria chordalis*, *Ulva* sp. and *Sargassum muticum* seaweeds when using EAE and traditional SLE [35]. In accordance with a study comparing EAE using cellulase, hemicellulose, and a mixture of both to *P. palmata* and *S. chordalis* while testing HHPE, showed that cellulase alone was generally more effective and further enhanced SP yield in combination with HHPE [36]. In other study, the use of cellulase-assisted extraction (using commercially available CellicCTec3^®^) on brown and red seaweeds, *Macrocystis pyrifera* and *Chondracanthus chamissoi,* led to significantly higher protein yields compared to SLE, 36.10% and 74.60% respectively (Table 2), as a result of the optimization of the extraction process through a central composite experimental design [37]. Other authors reported that a higher yield of alkaline soluble protein was recovered from *P. palmata* following treatment with a combination of commercial glucanase cocktails (Shearzyme and Celluclast) [22]. This resulted in a total protein recovery of 8.39% when compared to that obtained following OS and mechanical shear (6.77% and 6.92%, respectively). Therefore, EAE employing glucanases may achieve high protein yield, although the co-extraction of other components (i.e., phenolics) may also occur [25].

Besides glucanases, proteases are also used in EAE of SP, to achieve the release of proteins, peptides, and amino acids present in the supernatant of extracted samples [28].

### 2.3. Pulse-Electric Field Assisted Extraction

The use of electric pulses to break cell membranes and walls down has been growingly investigated to aid biomolecules’ extraction. The application of PEFs can help in protein extraction from macroalgae by generating high voltage (kV) electric pulses of different length, ranging between micro and milliseconds, which result in reversible or irreversible electroporation of cell wall and membrane [43]. PEF is considered as a rapid and effective green technology that should cope with the own limitations, since conductivity and electrode gap may limit the widespread application of PEF at a larger scale [44]. PEF has been extensively applied to improve protein extraction from green seaweed species. For example, protein extraction from *Ulva* sp. was optimized using OS combined with PEF followed by hydraulic press treatment, increased protein extraction from 2.25% to 5.38% (*w*/*v*) [45]. Similar protein yields were reported in the same genus when using PEF combined with hydraulic pressure. Another study reported the application of PEF with a custom-made insulated gate bipolar transistor-pulsed generator coupled with a gravitation press-electrode to aid protein extraction from *U. ohnoi*, improving protein yield from 3.16% to 14.94% [23]. PEF coupled with mechanical pressing was employed for protein extraction from *Ulva* sp. An optimized protocol resulted in a seven-fold increase in total protein yield (~20% protein in the extract) when compared to an extract obtained using an OS [23]. Moreover, PEF has been regarded as a workable method for SP extraction and simultaneous hydrolyzation, as extracted proteins by this method display higher antioxidant activity than non-PFE extracted ones.

### 2.4. High Hydrostatyc Pressure Extraction

High hydrostatic pressure (HHP) improves extraction efficiency because of the application of pressurized conditions (up to 1000 bar) to induce cellular disruption [43]. Factors affecting HHPE efficiency include the choice of solvent along with the operating pressure, temperature, and duration. HHPE is considered as an effective green extraction technology due to its short processing time, mild operating temperature, and high recovery yields. Therefore, this technique may be suitable for heat sensitive compounds, although pressure-induced conformation changes/denaturation of proteins may need to be taken into consideration. The application of HHP (600 MPa for 4 min) to aid protein extraction from two brown seaweeds, *F. vesiculosus* and *Alaria esculenta*, and two red seaweeds, *P. palmata* and *C. crispus* was investigated [24]. HHP treatment appeared to be the most effective for protein extraction from *F. vesiculosus* (23.70% of total protein). On the contrary, autoclave treatment yielded higher protein content (21.50%) than the HHP treatment (14.90%) in the case of *P. palmata* [24]. However, in the case of *S. chordalis*, HPP treatment (400 MPa for 20 min) only resulted in a 2.60% increase in protein yield [24]. The use of HHPE in combination with other extraction techniques has also been investigated, particularly HHP-assisted enzyme extraction. Interestingly, it has been reported that lower temperatures and higher pressures could yield lower proportion of undesirable artifacts, such as polyphenols, and higher protein yields [36]. HHP treatment (400 MPa for 20 min) alone did not increase protein yield from dried ground *P. palmata*, while HHP-assisted hydrolysis with cellulase and hemicellulase resulted in a 17% increase in protein yield compared to the control. Managing extraction parameters to lower polyphenol extraction would contribute to the optimization and enhancement of SP yield, although further research is still needed to assess HHPE as a workable, specific SP extraction method.

### 2.5. Ultrasound-Assisted Extraction

The use of UAE whether as sonication pretreatment or central component of the extraction process has gained significant interest in maximizing algal protein extraction. This technique cause cell wall breakdown by the acoustic cavitation phenomenon, in which the implosion of air bubbles results in the generation of a potent mechanical energy that disrupts the cell wall [46]. The main advantages of this extraction method are its short processing time, the independence of temperature and the use of water as the solvent of choice, which also plays a meaningful factor in the case of algal samples, thanks to their susceptibility to OS [39]. However, the application of high ultrasound power for extended periods of time can lead to an excessive heat production that may significantly alter protein structure. UAE can be used simultaneously with other techniques, such as OS and EAE. These integrated approaches have led to increasing protein extraction yields. Combination of UAE and EAE with a cellulase cocktail promoted a significantly higher phycobiliprotein yield from the red seaweed *Grateloupia turuturu*, compared to treatment with EAE alone [32] (Table 2). UAE using NaOH as solvent allowed for obtaining a 3.4% protein yield (*w*/*w*) from the red seaweed *N. haitanensis* in just 20 min of sonication at room temperature [38]. In addition, O’ Connor et al. proved that sonication combined with ammonium sulfate precipitation resulted in the highest protein recovery from *F. vesiculosus* and *Chondrus crispus*, with yields of 35.1% and 35.5% of total protein, respectively [24] (Table 2). This was compared to other approaches, like HHPE or laboratory autoclave treatment that yielded protein recoveries ranging from 16.1% to 24.3% out of total protein. Application of UAE as a food-grade extraction protocol at a larger scale has been previously proved in the protein extraction from *Ulva* sp. and *Gracilaria* sp., allowing to recover 70% and 86% of total proteins, respectively [39] (Table 2).

### 2.6. Microwave-Assisted Extraction

During the application of MAE, microwave energy is converted into heat by ion conduction and dipole rotation, and non-polar compounds are not heated [43]. Therefore, MAE may not be suitable for the extraction of heat-sensitive bioactive compounds. Although it has been recognized as an efficient low-energy extraction approach, MAE has to date been more widely used to extract carbohydrate or phenolic compounds rather than bioactive proteins/peptides from seaweed samples [40]. Microwave-derived heating and ionization can promote the release of intracellular components from the matrix into the extracting solution, partly because of the damage effect of microwave on cell wall and cell membrane. Because of the high ash content of edible seaweeds contributing to ionic conduction, MAE constitutes a powerful technique to be applied to macroalgae protein extraction. Compared with conventional extraction, MAE shortens extraction time and reduces solvent consumption, as reported for the application of acid/alkali SLE and MAE to *U. ohnoi*, where MAE achieved higher SP yield (23% dw) after 20 min, while SLE extraction required at least 24 h to yield a significant amount of protein from seaweeds [22] (Table 2). Most importantly, although SLE reached higher yields, in this study MAE was conducted in just 5 min and only with the aqueous SP fraction [40]. In summary, although MAE efficiency was reported to be lower than SLE in terms of SP yield, processing times can be reduced saving energy consumption and thus improving the efficiency of this technique [47].

## 3. Protein Purification

After extraction, SP should be purified to drop other co-extracted components, represented by polysaccharides, minerals, and phenolic compounds. Protein purification methods are based on molecular charge and size differences, and the most extensively used methods are ultrafiltration (UF), ionic-exchange chromatography (IEC), and dialysis.

### 3.1. Ultrafiltration

**Figure 1:**
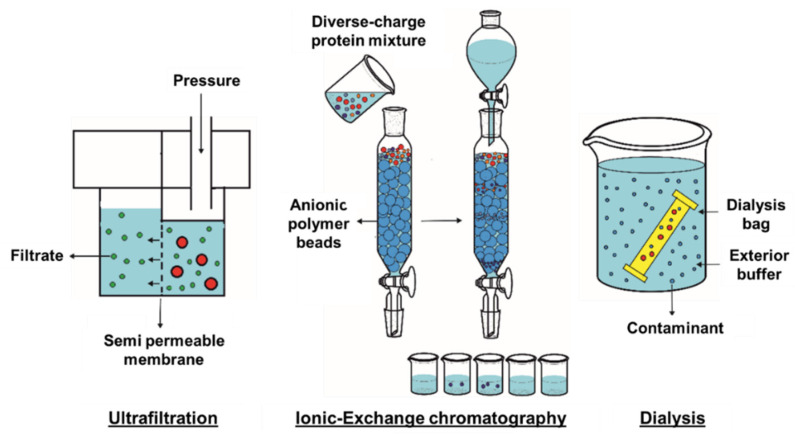
Depiction of ultrafiltration, ionic exchange chromatography and dialysis in protein purification.

UF is an important and widespread purification technique developed by membrane filtration and based on the separation of biological molecules according to their molecular mass [48,49]. The method is suitable for purifying proteins from low-mass contaminating molecules and can be used to concentrate protein solutions at the same time (Figure 1). UF membranes must supply great separation ability, high flux value, and good mechanical and chemical properties. The membrane carrier must be chemically inert with excellent mechanical properties. The device should provide an easy way to remove the deposited layer as well as easy washing and sanitation of the membrane [50]. By the action of pressure force, solvents and molecules of low molecular weight pass through the membrane, while larger molecules stay trapped. If finer separation is needed, the UF method needs to be coupled to more selective methods, such as chromatography or electrophoresis [48,51].

### 3.2. Ionic-Exchange Chromatography

Regarding its use on SP purification, this method is more extensively used in SP hydrolysates fractionation. Therefore, use of UF allows separating very low-molecular weight compounds from the extracted matrix and is especially useful for purifying BAPs after enzymatic hydrolysis of SP. Indeed, different studies used UF membranes of <1 kDa molecular weight for purifying an enzymatic hydrolysate of *Ulva* sp. and yielded fractions with greater anti-inflammatory potential and inhibitory capacity against the angiotensin I-converting enzyme (ACE) [52]. Use of UF to separate low-molecular weight peptides from *Ulva rigida* hydrolysates doubled the ACE inhibitory activity of the sample in comparison to non-filtered hydrolysate [53]. Therefore, this technique may prove useful for concentrating BAPs and SP hydrolysates.

IEC is widely used for purification of charged biomolecules such as proteins, peptides, or amino acids [54,55]. IEC presents a high performance, because it can be applied to a large number of different proteins using high-affinity and cost-effective buffers. The basis of IEC is the interaction of charged molecules flowing in the mobile phase with charged groups of the stationary phase (column packing matrix). Amino acids present charges of different polarity, for example lysine and arginine have a positive charge, while aspartic acid and glutamic acid are negatively charged at physiological pH [56]. Thus, each protein has variable net charges based on its aminoacidic composition but also depending on the pH of the solution they are dissolved in. Thus, the proteins’ IP is key to purify these by IEC, since it will find the best initial conditions for protein purification. Ion exchangers are divided into two classes: strong and weak. Strong ion exchange ligands keep their charge characteristics and ion exchange capacity in a wide range of pH, while weak ion exchange ligands show a more pronounced change in their exchange capacity with pH modifications [57]. If purification is conducted at a pH above 9 for anion exchange or below 6 for cation exchange, then a strong ion exchanger must be used. However, if purification is performed at less extreme pH values, then both strong and weak ion exchangers should be used, and the obtained results should be compared to optimize the purification process. Protein binding and elution is based on the competition between the charged amino acid groups on the protein surface and the charged conditions in the binding buffer for the oppositely charged groups in the stationary phase (Figure 1).

The protein sample is applied to an ion exchange column in a low salt solution [55,56,57,58]. The charged groups on the protein have a high binding affinity for the charged counterions on the ion exchange resin. Different affinity columns may be used to keep proteins and peptides, depending on their functional group. For example, sulfopropyl functional groups, as strong cation exchangers may keep molecules on a range of pH 4 to 13. Conversely, diethylaminoethyl is a weak anionic exchanger and may retain molecules in pH from 2 to 9 [59]. Regarding the use of this technique in SP, the most commonly used columns are anionic exchangers [60]. For example, IEC allowed for accurate fractionation of anticoagulant peptides from a *Neopyropia yezoensis* hydrolysate [61].

### 3.3. Dyalisis

Dialysis is a common method for removing contaminants by selective and passive diffusion through a semipermeable membrane, such as a dialysis tube. Dialysis is most used to remove small molecules in solution, such as salts, reducing agents, or preservatives, among others. This technique is a simple but time-demanding method since separation depends on diffusion rate. The dialysis system consists of a tube, which holds a semipermeable membrane, e.g., porous cellulose, closed on both sides to contain a protein solution [62]. The tube is immersed in a much larger vessel filled with buffer (Figure 1). Low-molecular weight particles will diffuse through the semipermeable membrane, while protein molecules, due to their larger size, will remain inside the tube. On the other hand, the buffers inside and outside the dialysis tube will change during diffusion until equilibrium is reached. This equilibrium obeys the Donnan effect, which keeps electrical neutrality on both sides of the membrane [63]. The process of diffusion of small molecules through the membrane is accompanied by the increased movement of protein molecules inside the dialysis tube. As they cannot diffuse due to their size, proteins remain inside the membrane. It should be noted that polyvalent proteins do not allow charged particles to move away from the membrane surface, thus preventing them from setting up an equilibrium potential [64]. On the other hand, the Donnan effect has a significant effect on the colligative properties of ions in the case of high concentrations of proteins or buffers with low ionic strength. Finally, during the dialysis process, the buffer inside the dialysis tube is gradually replaced with the solution in the outer vessel. In this way, efficient removal of small contaminants from the tube is achieved [48]. In summary, dialysis is indeed one of the most used techniques for separating peptides and proteins from natural matrixes. Hence, pairing high-yield extraction techniques with dialysis allows obtaining SP isolates with greater protein content compared to other purification methods [24,65].

## 4. Hydrolysis and Peptide Production

**Figure 2:**
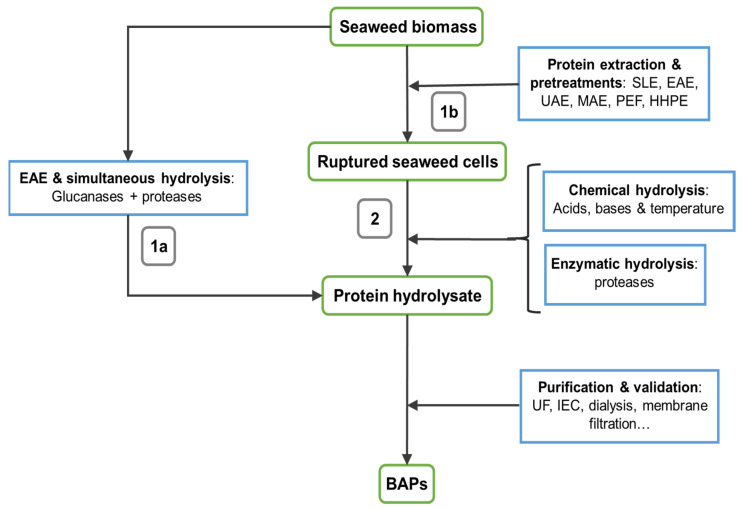
Schematic depiction of production of SP hydrolysates and bioactive peptides (BAPs) from seaweed. Proteins are extracted from seaweed cells and subsequently hydrolyzed in a single (1a) or multiple steps (1b and 2) to produce a protein hydrolysate. The protein hydrolysate is further separated into peptide fractions using diverse purification methods.

Protein hydrolysis allows producing protein hydrolysates and BAPs from seaweed. Figure 2 schematically depicts the steps involved in seaweed BAPs production. As mentioned above, pre-treatment strategies may be used to both simplify the extraction process and enhance hydrolyzed SP yield. While some SP hydrolysates have been reported to be biologically active, SP-derived BAPs have gained much attention due to their higher bioactive potential. Hence, once extracted and isolated, SP need to be hydrolyzed into smaller peptide sequences to become biologically active [66]. Hydrolysis can be done either chemically or using proteases. Chemical hydrolysis is typically performed using acids and/or alkali at temperatures over 40 °C. While the procedure is simple and inexpensive, the composition of the hydrolysate is difficult to control, also yielding products with modified amino acids in their peptide sequence [67]. For example, strong organic acids can efficiently hydrolyze hydrophobic peptide bonds, as a mixture of HCl and trichloroacetic acid was found to destroy tryptophan [68]. Conversely, alkaline hydrolysis with NaOH can reduce cystine, arginine, threonine, serine, and isoleucine; form unusual amino acid residues such as lysinoalanine or lanthionine; as well as induce isomerization of L-lysine residues into D-lysine ones [69]. Similarly, high temperatures may be used to induce hydrolyzation, without need of aiding chemicals [42].

In contrast, enzymatic hydrolysis is performed at lower temperature in order to preserve the native structure of the used protease conducting the hydrolysis process. In addition, enzymatic hydrolysis is more specific to the desired peptides and results in higher peptide yield. Due to the lack of organic solvents, the purification steps are less labor-intensive and side reactions are less pronounced [70,71]. The experimental conditions of enzymatic hydrolysis are typically governed by the needs of the enzyme. The process can be done in a single or, at times, in multiple steps [72]. Substrate-to-enzyme ratio as well as the duration of the hydrolysis have a significant effect on the nature and yield of the final products [70]. Table 3 illustrates a range of bioactive peptides obtained by enzymatic hydrolysis from seaweed with the aid of a number of proteases. The most extensively used proteases to perform protein hydrolysis are trypsin, pepsin, papain, and α-chymotrypsin. They are used both individually and in combination with other proteases. More recently, commercially available enzyme cocktails including proteases and carbohydrases are used (Table 3).

A range of experimental conditions (temperature, pH, agitation speed, enzyme-to-substrate ratio, hydrolysis time) need to be optimized for best bioactivity. Typically, a mixture of peptides is obtained, of which only a few are biologically active. The active ones are typically isolated from the mixture using membrane filtration and reverse-phase chromatography techniques [90,91]. SP bioactive peptides range in sizes from 2 to 20 amino acids with the cut-off masses usually ranging from 5 kDa to 30 kDa [92]. Nonetheless, as the nature of SP varies for each seaweed, the resulting BAP profile will also be different depending on the hydrolysis conditions and protease of choice, as these account for different cut-off points [93]. The use of pepsin or trypsin may be also useful to obtain insight on in vivo SP degradation and potential bioavailability of BAPs. Indeed, a wide range of commercially available enzymes has been evaluated to screen out for the possible BAPs produced, such as Flavourzyme, Corolase, or fungal and bacterial proteases [75,76]. The type of the chosen proteolytic enzyme has been shown to have impact on the resulting bioactivity, as it defines the resulting BAP profile. In a recent study, *Gracilariopsis lemaneiformis* proteins were hydrolyzed by different proteases such as trypsin, pepsin, papain, α-chymotrypsin, or Alcalase and hydrolysates obtained by α-chymotrypsin hydrolysis displayed the highest antioxidant activity [81]. Studies performed by Fitzgerald et al. and Harnedy et al. proved that using papain or Corolase yielded highly different BAP in *P. palmata* protein isolates hydrolysis [73,75]. Hydrolysis is stopped by enzyme denaturation, often achieved with pH shifts or heating. One disadvantage of thermal deactivation is the fact that higher temperatures might speed up the kinetics of unwanted processes. An approach to overcome this problem is enzyme immobilization on solid substrates. The use of immobilized enzymes enables the enzymes to be separated from the hydrolysis mixture by filtration instead of increased temperature. An additional advantage is that immobilized enzymes are prevented from being aggregated with the peptides [94]. One challenge of enzymatic hydrolysis is the fact that the algal proteome needs to be known prior to hydrolysis so that enzymes with according cut sites are used. To improve the purity and yield of BAPs, enzymatic hydrolysis is often preceded by physical cell disruption methods, including PEF and bead milling [95,96]. Nonetheless, the hydrolytic activity of the chosen protease can be inhibited by co-extracted compounds such as polysaccharides or phenolic compounds, and optimization studies must be conducted [82,97]. Hydrolysates may be purified by the above-mentioned techniques to ensure concentration of lower molecular weight peptides or to obtain purified BAPs. In this sense, sequential purification processes seem to work best for obtaining high-purity peptides [39].

## 5. Bioactive Properties and Applications of Seaweed Proteins and Derived Products

As mentioned throughout this work, proteins and derived products obtained from seaweed show various biological activities, as reported by in vitro and a few in vivo tests, such as antioxidant, antimicrobial, anti-inflammatory, antihypertensive, or even antitumor [1]. Regarding SP, the extent of their displayed bioactivities varies upon protein composition, season, but also hydrolysis degree and purification methods applied. The three main bioactivities consistently reported for SP and BAPs from different species are antioxidant, anti-inflammatory and antihypertensive [97,98]. In vitro antioxidant activity is usually tested against oxidation-sensible molecules, such as 2,2-diphenyl-1-picrylhydrazyl (DPPH) or 2,2’-Azino-bis(3-ethylbenzothiazoline-6-sulfonic acid) (ABTS). It has been reported that SP derived peptides display higher antioxidant activity than SP hydrolysates [76]. These could be due to the concentration and better accessibility of released amino acids from folded proteins. Concentrated 1 < kDa BAPs from *P. dioica* hydrolysates showed significant antioxidant activity (20.88 µmol Trolox equivalents) in a wide range of assays, including DPPH and ABTS [78]. Comparing results with free aminoacidic composition of the isolated fractions, the authors suggested that higher presence of sulfated amino acids, like cysteine or methionine, could be related to these results, as proved by these in vitro assays. Different results were reported for *Macrocystis pyrifera* and *Chondracanthus chamissoi* BAPs, accounting for 193 and 167 µmol Trolox equivalents respectively, in a DPPH assay [37]. Antioxidant assays with *P. palmata* hydrolysates also suggested that a higher protein hydrolysis degree was related to higher antioxidant activity [93].

Anti-inflammatory assays are often conducted on cell culture tests, assessing production or inhibition of pro-inflammatory mediators, such as interleukins. SP hydrolysates produced using several proteases from *Ulva* sp. were found to exert significant anti-inflammatory activity by upregulating interleukin 10 and inhibiting tumor necrosis factor alpha (TNF-α) expression [52]. Hydrolysates obtained from *Pyropia columbina* also displayed anti-inflammatory properties in vitro with similar tested mechanisms, while also inhibiting expression of necrosis factor κB [77].

Yet, the most well studied bioactive property of SP, derived hydrolysates, and BAPs is their hypotensive activity [86]. These properties may be assessed in vitro by checking the inhibition capacity of these molecules against key cardiac tension enzymes such as ACE, being the most extensively used model, but also renin or endothelin I in a lesser extent [86]. Conversely, in vivo tests may be conducted monitoring blood pressure and cardiac rhythm in animal models. The previously mentioned study of Vasquez et al. showed that the *M. pyrifera* extract displayed an ACE inhibition of 38.8% compared to control and that the smaller the size of the purified peptides, the greater inhibition degree was [37]. However, *C. chamissoi* extracts did not show a detectable ACE-inhibitory activity. Authors proposed that this could be due to interactions between co-extracted compounds and ACE enzymatic reaction products [37]. Protein hydrolysates from *P. palmata*, showed that ACE inhibitory activity was independent from the time of harvesting, but was related to its hydrolysis degree [93]. Nine ACE-inhibitory peptides derived from phycobiliproteins were found, and the oligopeptide LRY demonstrated particularly high inhibitory activities, with an IC_50_ of 0.044 mM [99]. Similarly, the peptide PAFG, obtained from *U. clathrata* protein, showed an IC_50_ value of 35.9 mM on ACE-inhibitory activity [83] (Table 3). Moreover, since BAPs are obtained through different hydrolysis methods, these have shown to be stable towards gastrointestinal proteases like pepsin or trypsin, as well as the acidic pH of digestive reactions [37,83].

Other reported bioactivities include antidiabetic or antimicrobial, associated to phycobiliproteins and lectins, but also some SP hydrolysates [100]. Antidiabetic activity was characterized in *P. palmata* hydrolysates through in vitro inhibition of dipeptidyl peptidase IV (DPP-IV). Results showed that Corolase-hydrolyzed SP accounted for a much significant DPP-IV inhibition (IC_50_ = 1.65 mg/mL) [93]. BAPs from *Saccharina longricuris* displayed antimicrobial activity against several bacterial pathogens, such as *Escherichia coli*, *Staphylococcus aureus*, or *Pseudomonas aeruginosa*, achieving a 40% growth inhibition at 2.5 mg peptides/mL [88].

**Figure 3:**
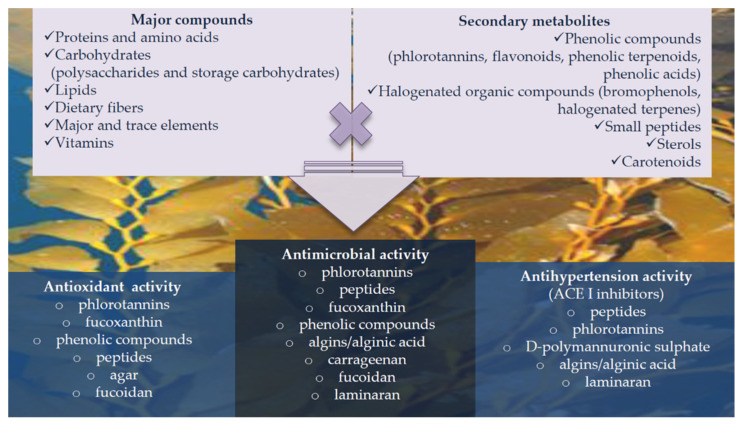
Reported bioactivities of different seaweed biomolecules with potential industrial application.

Therefore, based on their reported bioactivities, these macroalgae molecules may be used for diverse industrial applications (Figure 3). 

Both antioxidant and antimicrobial properties make SP hydrolysates a desirable choice for their use as food additives, preservatives, and protein fortifiers. Additionally, the ACE-inhibitory and anti-inflammatory activities widely apparent in several SP, could make them to be assessed as nutraceuticals, functional ingredients for food and feed, or cosmetic ingredients [101]. The fact that this effect has been assessed in vivo, with *Undaria pinnatifida* BAPs allowing for reduced blood pressure in rats, could be argued to also be the basis for developing new hypotensive pharmaceuticals [42,87]. Moreover, SP hydrolysates keep their bioactive properties when combined with other food matrices [17,102]. In the same way, the antioxidant properties attributed to BAPs make them excellent natural ingredients for cosmetic applications, as SP also hold significantly higher levels of taurine compared to other vegetal sources [91]. In summary, the full set of already reported bioactivities and the ongoing research to fully characterize the properties and production of SP and derived molecules will unveil the full extent of the potential applications of these natural molecules.

Development of such applications would thus confer an added value to this underexploited natural resource. Furthermore, these properties are also well accounted for co-extracted bioactive compounds in SP extractions, such as cell wall polysaccharides (e.g., carrageenan, fucoidan…) or phenolic compounds, such as brown seaweeds phlorotannins [103]. Altogether, seaweed hold several different bioactive molecules with similar reported activities, that in the case of some extracts, could act in cooperation [104] Thus, SP could be considered as part of the whole composition of health-promoting compounds of seaweeds and their extracts be applied for a wide diversity of applications [105] (Figure 3).

## 6. Conclusions

SP and especially their hydrolysates and several identified BAPs, have been repeatedly reported to show significant bioactivities such as antioxidant or anti-inflammatory, but most importantly, antihypertensive. Their bioactivities raise potential applications in food and animal feed, whether as functional additives or colorants in the case of phycobiliproteins. On the other hand, BAPs may find use as cosmetic ingredients with antioxidant activity, while thanks to their antihypertensive activity, they can be used as nutraceuticals in the pharmaceutical industry. Nonetheless, these seaweed components need more extensive and in-depth research to fully characterize their bioactive properties, production methods, and potential in vivo effects.

## Figures and Tables

**Table 1:** Average protein content, essential amino acidic composition, and digestibility of few representative seaweed sources.

Species	Protein (% dw)	EAA Composition (% TAA)	EAA (% prot.)	Digestibility	Reference
**Rhodophyta**					
*Gracilaria gracilis*	31–45%	R 1.3, H 0.2, K 1.6, T 1.7, I 2.3, L 1.9, V 3.1, M 0.2, F 1.7, C 0.4, P 1.0, A 1.9, Y 1.3, D 2.6, E 2.4, G 1.1, S 1.6	14%	68% (in vivo)	[8,10]
*Palmaria palmata*	55%	T 4.1, V 5.4, M 2.0, I 4.3, L 7.2, K 5.7, F 4.7, W 0.9, H 1.7, S 6.2, Q + E 14.9, P 7.9, G 5.8, A 8.8, C 2.5, D + N 9.7, Y 2.7, R 5.6	36%	56% (pancreatin)	[8,11]
*Porphyra* sp.	31%	D 8.5, T 5.3, S 4.9, E 10.2, G 5.1, A 6.2, V 5.2, I 3.3, L 5.9, Y 3.4, F 3.5, H 2.6, K 5.2, R 5.9, P 3.6, C 1.3, M 1.8, W 0.7	51%	57% (pepsin), 56% (pancreatin), 78% (pronase)	[8,12]
**Clorophyta**					
*Cladophora rupestris*	12%	A 5.5, R 6.5, N 15.3, E 15.3, G 6.7, H 1.4, I 3.6, L 7.0, K 7.4, M 1.8, F 4.5, P 5.7, S 4.3, T 5.1, Y 4.3, V 5.8	13.9%	N.A.	[13]
*Codium fragile*	11%	D 0.8, E 1.1, S 0.5, H 0.1, G 0.5, T 0.6, R 0.4, A 0.6, Y 0.4, V 1.4, M 0.9, C 0.1, I 0.4, L 0.7, F 0.5, L 0.5	5.4%	N.A.	[14]
*Ulva* sp.	27%	D 1.5, E 1.5, S 0.8, H 0.1, G 0.8, T 0.8, R 0.5, A 1.1, Y 0.4, V 0.3, M 0.7, I 0.5, L 1.0, F 1.2, K 0.7	12%	17% (pepsin), 67% (pancreatin), 95% (pronase)	[8,15]
**Ochrophyta**					
*Fucus serratus*	4%	A 6.8, R 4.4, N 14.0, E 1596, G 5.8, H 1.7, I 4.0, L 6.8, K 5.5, M 1.9, F 5.0, P 4.0, S 5.6, T 5.5, Y 3.7, V 5.6	4.6%	N.A.	[13]
*Sargassum fusiformis*	12%	D 9.1, T 4.1, S 5.6, E 18.7, G 4.8, A 4.3, V 4.9, I 4.0, L 6.7, Y 2.8, F 4.6, H 2.6, K 3.1, R 4.5, P 3.8, C 0.9, M 1.6, W 0.4	10.9%	N.A.	[12]
*Undaria pinnatifida*	19.8%	D 8.7, T 4.4, S 4.0, E 14.5, G 5.1, A 4.7, V 5.2, I 4.1, L 7.4, Y 2.9, F 4.7, H 2.5, K 5.6, R 5.2, P 3.6, C 0.9, M 1.7, W 0.7	35.5%	24% (pepsin), 48% (pancreatin), 87% (pronase)	[8,12]

Abbreviations: dw: Dry weight, N.A: Not analyzed, prot: Protein, EAA: Essential amino acids, TAA: Total amino acids.

**Table 2:** Source, protein yield, pre-treatment, extraction, and purification methods described. Protein yields are indicated as % of algal biomass dw.

Source	Pretreatment	Extraction Method	Precipitation and Purification	Yield (% dw)	Reference
**Rhodophyta**					
*Palmaria palmata*	Freeze-dried OS, (1: 20), 16 h, 4 °C // EPr, Celluclast + Shearzyme (E:S 4.8 × 10^3^ U/100 g), pH 5, 24 h, 40 °C	SLE ak, 0.12 M NaOH + 0.1 mg/L NAC, 1 h, 25 °C	Pr: IP, pH 4, 1N HCl	11.57%	[22]
	Freeze dried	EAE (E:S 0.5) Celluclast 0.2% + Alcalase 0.2%, pH 4.5, 14 h, 50 °C // SLE ak, 0.1 M NaOH + 1 g/L NAC, 1.5 h, 25 °C	Pr: IP, pH 3, 5M HCl	13.7%	[28]
	Dried, milled Hydrated (6%) Tris-HCl, pH 5, 16 h, 4 °C	EAE-HHPE, Hemicellulase (E:S 0.05), pH 4.5, 400 MPa, 20 min 40 °C	-	6.3%	[36]
*Soliera chordalis*	Dried, milled Hydrated (6%) Tris-HCl, pH 5, 16 h, 4 °C	EAE-HHPE, Hemicellulase (E:S 0.05), pH 4.5, 400 MPa, 20 min, 40 °C	-	3.4%	[36]
*Porphyra dioica*	Freeze dried OS, (1: 20), 16 h, 4 °C	SLE ak, NaOH 0.12 M, 1 h, 25 °C	Pr: IP, pH 4.5 1M HCl	14.28%	[34]
*Neoporphyra haitanensis*	Freeze dried	UAE ak, 400 W, 40 kHz, 0.01% NaOH, 20 min, 35 °C	Pr: (NH_4_)_2_SO_4_, 40%, 4 h, 4 °C	3.8%	[38]
*Chondrus crispus*	Freeze dried	UAE, _d_W (1:20), 42 Hz, 1 h, 4 °C	Pr: (NH_4_)_2_SO_4_, 80%, 1 h, 4 °C Pu: DI, 3.5 kDa	~6.7%	[24]
	Freeze dried	HHPE, _d_W (1:20) 600 MPa, 4 min, 4 °C	Pu: Filtered, 100 μm nylon bag	~3.1%	
*Condracanthus chamissoi*	Oven dried (60 °C) 0.1 M NaOAc buffer, pH 4.5, 10 min, 50 °C	EAE Cellic CTec3 (E:S 0.1, 1.64 U/mg), pH 4.5, 16 h, 50 °C	Pr: Cold acetone (1: 4), 2 h	6.35%	[37]
**Clorophyta**					
*Ulva* sp.	Oven dried (60 °C), freeze dried, milled	UAE ak (2×), (1:10), 1M NaOH, sonication (Hz non specified), 2 h, 25 °C	Pu: Filtered (0.45 μm) // DI, 2 kDa // IEC, Tris buffer, pH 9.5 // DI 2 kDa	5.4%	[39]
	Freeze dried, milled	SLE (1:20), lysis solution (8 M urea, 2% Tween, 1% PVP, 30 mM DTT), 16 h, 4 °C	Pu: DI, 6–8 kDa, 4 °C, 16 h	11.88%	[31]
	Untreated	PFE aq, _d_W, 50 kV, 50 pulses, 0.5 Hz, 34 kJ // Mechanical press	Pr: DI, 100–500 kDa	4.7%	[23]
*Ulva ohnoi*	Oven dried (55 °C), milled	SLE aq, _d_W (1: 20), 16 h, 30 °C // SLE 1M NaOH, pH 12, 30 °C, 2 h	Pr: IP, pH 2.25, 10% *v*/*v* HCl	12.28%	[27]
	Fresh, pulped	SLE aq, _d_W (1:20), 16 h, 30 °C // Filtration (100 μm) // SLE 1M NaOH, pH 12, 30 °C, 2 h // Filtration (100 μm)	Pr: IP, pH 2.25, 10% *v*/*v* HCl	17.13%	
	OS (1:10), 30 min, 40 °C // 0.05M HCl, 1 h, 85 °C	MAE aq, _d_W (1:34), 5 min., 123 °C	Pr: IP, pH 2.25, 10% *v*/*v* HCl	11.3%	[40]
*Ulva compressa*	Oven dried (60 °C), milled OS (1: 20), 16 h, 35 °C	SLE ak, 1M NaOH, pH 12 + 0.5% 2-mercaptoethanol, 2 h, 25 °C	Pr: (NH_4_)_2_SO_4_ 80% Pu: DI (kDa n.s.)	6.48%	[41]
**Ochrophyta**					
*Ascophyllum nodosum*	Oven dried (40 °C) UPr, _d_W (1: 20), 750 W, 20kHz, 10 min, 4 °C	SLE ak (1:15) 0.4M NaOH 1 h, 4°C // SLE ac (1:15) 0.4M HCl 1 h, 4 °C	Pu: HPSEC, 150–300 Å, 15 min, 40 °C	4.23%	[33]
*Alaria esculenta*	Freeze dried, milled	HAE aq Autoclave, _d_W (1:20), 0.101 MPa, 2 × 15 min, 124 °C	Pu: Filtered, 100 μm muslin bag	~2.4%	[24]
*Sargassum patens*	Freeze dried OS, (1:20), 16 h, 35 °C	SLE ak, 1M NaOH, pH 12 + 0.5% 2-mercaptoethanol, 2 h, 25 °C	Pr: (NH_4_)_2_SO_4_ 85% Pu: DI (kDa n.s.)	8.2%	[26]
*Macrocystis pyrifera*	Oven dried (60 °C) 0.1 M NaOAc buffer, pH 4.5, 10 min, 50 °C	EAE Cellic CTec3 (E:S 0.1, 1.64 U/mg), pH 4.5, 16 h, 50 °C	Pr: Cold acetone (1:4), 2 h	7.39%	[37]
*Undaria pinnatifida*	Dried, powdered	SLE aq, _d_W (1:3), 20 min, 93 °C	Pu: HPLC, Develosil ODS-5 column, 25% CH_3_CN + 0.05% CF_3_COOH	12%	[42]
*Fucus vesiculosus*	Freeze dried, milled	UAE aq, _d_W (1:20), 42 Hz, 1 h, 4 °C	Pr: (NH_4_)_2_SO_4_, 80%, 1 h, 4 °C Pu: DI 3.5 kDa	~1.8%	[24]

Abbreviations: OS: Osmotic shock, EPr: Enzymatic pretreatment, Upr: Ultrasonic treatment, //: sequential procedures, SLE: Solid-liquid extraction, aq: Aquose, ak: Alkaline, ac: Acid, Pr: Precipitation, Pu: Purification, _d_W: Deionized water, DI: Dialysis, NAC: N-acetyl-L-cysteine, EAE: Enzyme-assisted extraction, UAE: Ultrasound-assisted extraction, HHPE: High hydrostatic pressure extraction, PEF: Pulse-electric field, PBS: Sodium phosphate buffer, PVP: polyvinyl propylene, DTT: dithiothretol, IEC: Ionic exchange chromatography, HPSEC: High performance size exclusion chromatography, IP: Isoelectric precipitation. n.s.: Not stated.

**Table 3:** Bioactive SP-derived hydrolysates, peptides, aminoacidic sequence of bioactive peptides, and proteolytic methods used.

Seaweed	Hydrolysis Method	Peptide Sequence	Bioactivity Reported	Reference
***Rhodophyta***				
*Palmaria palmata*	Papain, (E:S 20.7), pH 6, 24 h, 60 °C	IRLIIVLMPILMA, NIGK, IR	Renin, DPP IV, PAF-AH inhibition	[65,73,74]
	Corolase PP (E:S 1), pH 7, 2 h, 50 °C	ILAP, LLAP, MAGVDHI, FITDGNK., NAATIIK, ANAATIIK, SDITRPGGQM, DNIQGITKPA., LITGA., LITGAA., LITGAAQA., LGLSGK., LTLAPK, LTIAPK, ITLAPK ITIAPK, VVPT, QARGAAQA	Antioxidant, DPP IV inhibition	[75,76]
*Pyropia columbina*	Fungal protease concentrate (E: S 5) pH 4.3, 3 h, 55 // Flavourzyme (E:S 2), pH 7, 4 h, 55 °C	N.A.	Antitumor, anti-inflammatory, antioxidant	[77]
*Neopyropia yezoensis*	Pepsin (E:S 0.025), 5 h, 45 °C	NMEKGSSSVVSSRM	Anticoagulant	[61]
*Porphyra dioica*	Alcalase + Flavourzyme (E:S 1), pH 7, 4 h, 50 °C	DYYLR, AGFY, YLVA, AFIT, SFLPDLTDQ, MKTPITE, TYIA, LDLW	ACE, DPP IV inhibition	[34]
	Prolyve 1000, (E:S 1), 2 h, 50 °C	N.A. (higher < 1 kDa peptides proportion)	Antioxidant	[78]
*Porphyra* sp.	Pepsin (E:S 8), pH 2, 4 h, 37 °C	GGSK, ELS	α-amylase inhibition	[79]
*Mazzaella japonica*	Thermolysin (E:S 1), pH 7, 5 h, 37 °C	YRD, VSEGLD, TIMPHPR, GGPAT, SSNDYPI, SRIYNVKSNG, VDAHY, YGDPDHY, NLGN, DFGVPGHEP	ACE inhibition	[80]
*Grateulopia lemaneiformis*	α-chymotrypsin (E:S 4), pH 8, 2 h, 37 °C	ELWKTF	Antioxidant	[81]
	Trypsin (E:S 4), pH 8, 8 h, 37 °C	QVEY	ACE inhibition	[82]
***Chlorophyta***				
*Ulva*°C *lathrata*	Alcalase (E:S 5), pH 7.6, 90 min, 25 °C // 10 min, 100 °C	PAFG	ACE inhibition	[83]
*Ulva. intestinalis*	Trypsin + Pepsin + Papain (E:S 4), pH 8.42, 5 h, 28.5 °C	FGMPLDR, MELVLR	ACE inhibition	[84]
*Ulva rigida*	Pepsin (E:S 1), pH 2, 20 h, 37 °C // Bromelain (E:S 1), pH 7, 20 h, 37 °C	IP, AFL	ACE, Renin inhibition	[53]
*Ulva lactuca*	Papain (E:S 1), pH 6, 24 h, 60 °C	Total of 58 non-allergenic, ACE inhibitory peptides identified	ACE inhibition	[85]
*Ulva* spp	Purazyme + Flavourzyme // Alkaline protease-Protex 6L + Flavourzyme	N.A.	Anti-inflammatory (IL10 expression & TNF-α inhibition)	[52]
***Ochrophyta***				
*Sargassum maclurei*	Pepsin (80 U/g S), pH 2, 2 h, 37 °C // Papain (60 U/g S), pH 7, 3 h, 50 °C	RVLSAAFNTR, IMNILEK, GGVQAIR, KAALMEK, GVFDGPCGT, SGVFDGPCGT, QNIGDPR, AYSSGVSFK, RWDISQPY, LVYIVQGR, KPGGSGR, LGLSAKNYGR, KEAWLIEK, REVADDK, ENFFFAGIDK, QEMVDK, EEEEEEQQQ	Antyhypertensive (ACE & ET-1 inhibition)	[86]
*Undaria pinnatifida*	Protease S “Amano” (E:S 0.01), pH 8, 18 h, 70 °C	VY, IY, AW, FY, VW, IW, LW	Antihypertensive (ACE inhibition & in vivo)	[87]
	_d_W (3:20), 20 min, 93 °C	YH, KY, FY, IY	Antihypertensive (ACE inhibition & in vivo)	[42]
*Saccharina longicruris*	Trypsin (E:S 0.05), pH 7, 24 h, 30 °C	TITLDVEPSDTIDGVK, ISGLIYEETR, MALSSLPR, ILVLQSNQIR, ISAILPSR, IGNGGELPR, LPDAALNR, EAESSLTGGNGCAK, QVHPDTGISK	Antimicrobial	[88]
*Saccharina japonica*	Alcalase + Papain + Trypsin (E:S n.s.), time n.s., pH 7.5, 55 °C	KY, GKY, STKY, AKY, AKYSY, KKFY, FY, KFKY	ACE inhibition	[89]

Abbreviations: //, Subsequent procedures; n.s., Not stated; N.A.: Not analyzed; DPP, Dipeptidyl peptidase; PAF-AH, Platelet activating factor acetylhydrolase; ACE, Angiotensin 1 converter enzyme; ET-I, Endothelin-1; _d_W, Deionized Water.

## Data Availability

Not applicable.

## Abbreviations

SP	Seaweed proteins
dw	Dry weight
SLE	Solid–liquid extraction
HAE	Heat-assisted extraction
EAE	Enzyme-assisted extraction
UAE	Ultrasound-assisted extraction
MAE	Microwave-assisted extraction
PEF	Pulse-electric field
HHP	High-hydrostatic pressure
HHPE	High-hydrostatic pressure extraction
HAE	Heat-assisted extraction
EAE	Enzyme-assisted extraction
UAE	Ultrasound-assisted extraction
MAE	Microwave-assisted extraction
IP	Isoelectric point
Pr	Precipitation
Pu	Purification
DI	Dialysis
OS	Osmotic shock
Epr	Enzymatic pretreatment
Upr	Ultrasound pretreatment
_d_W	Deionized water
AK	Alkaline
MAAs	Mycosporine-like amino acids
EAA	Essential amino acids
GRAS	Generally recognized as safe
RAAS	Renin–angiotensin–aldosterone system
IC_50_	Half inhibitory concentration
DPP-IV	Dipeptidyl peptidase IV
MCF-7	Human breast cancer cell line
HepG2	Human liver cancer cell line
SGC-7901	Human gastric cancer cell line
A549	Human lung cancer cell line
HT-29	Human colon cancer cell line
UF	Ultrafiltration
IEC	Ionic-exchange chromatography
BAP	Bioactive peptide
DPPH	2,2-diphenyl-1-picrylhydrazyl
ABTS	2,2’-Azino-bis(3-ethylbenzothiazoline-6-sulfonic acid)
ACE	Angiotensin I converter enzyme
